# Phased gap-free genome assembly of octoploid cultivated strawberry illustrates the genetic and epigenetic divergence among subgenomes

**DOI:** 10.1093/hr/uhad252

**Published:** 2023-11-27

**Authors:** Yanhong Song, Yanling Peng, Lifeng Liu, Gang Li, Xia Zhao, Xu Wang, Shuo Cao, Aline Muyle, Yongfeng Zhou, Houcheng Zhou

**Affiliations:** National Key Laboratory for Germplasm Innovation & Utilization of Horticultural Crops, Zhengzhou Fruit Research Institute, Chinese Academy of Agricultural Sciences, Zhengzhou, 450009, China; National Key Laboratory of Tropical Crop Breeding, Shenzhen Branch, Guangdong Laboratory of Lingnan Modern Agriculture, Key Laboratory of Synthetic Biology, Ministry of Agriculture and Rural Affairs, Agricultural Genomics Institute at Shenzhen, Chinese Academy of Agricultural Sciences, Shenzhen 518120, China; National Key Laboratory for Germplasm Innovation & Utilization of Horticultural Crops, Zhengzhou Fruit Research Institute, Chinese Academy of Agricultural Sciences, Zhengzhou, 450009, China; National Key Laboratory for Germplasm Innovation & Utilization of Horticultural Crops, Zhengzhou Fruit Research Institute, Chinese Academy of Agricultural Sciences, Zhengzhou, 450009, China; National Key Laboratory for Germplasm Innovation & Utilization of Horticultural Crops, Zhengzhou Fruit Research Institute, Chinese Academy of Agricultural Sciences, Zhengzhou, 450009, China; National Key Laboratory of Tropical Crop Breeding, Shenzhen Branch, Guangdong Laboratory of Lingnan Modern Agriculture, Key Laboratory of Synthetic Biology, Ministry of Agriculture and Rural Affairs, Agricultural Genomics Institute at Shenzhen, Chinese Academy of Agricultural Sciences, Shenzhen 518120, China; National Key Laboratory of Tropical Crop Breeding, Shenzhen Branch, Guangdong Laboratory of Lingnan Modern Agriculture, Key Laboratory of Synthetic Biology, Ministry of Agriculture and Rural Affairs, Agricultural Genomics Institute at Shenzhen, Chinese Academy of Agricultural Sciences, Shenzhen 518120, China; CEFE, University of Montpellier, CNRS, EPHE, IRD, Montpellier 34000, France; National Key Laboratory of Tropical Crop Breeding, Shenzhen Branch, Guangdong Laboratory of Lingnan Modern Agriculture, Key Laboratory of Synthetic Biology, Ministry of Agriculture and Rural Affairs, Agricultural Genomics Institute at Shenzhen, Chinese Academy of Agricultural Sciences, Shenzhen 518120, China; National Key Laboratory of Tropical Crop Breeding, Tropical Crops Genetic Resources Institute, Chinese Academy of Tropical Agricultural Sciences, Haikou 570000, China; National Key Laboratory for Germplasm Innovation & Utilization of Horticultural Crops, Zhengzhou Fruit Research Institute, Chinese Academy of Agricultural Sciences, Zhengzhou, 450009, China

## Abstract

The genetic and epigenetic mechanisms underlying the coexistence and coordination of the four diverged subgenomes (ABCD) in octoploid strawberries (*Fragaria × ananassa*) remains poorly understood. In this study, we have assembled a haplotype-phased gap-free octoploid genome for the strawberry, which allowed us to uncover the sequence, structure, and epigenetic divergences among the subgenomes. The diploid progenitors of the octoploid strawberry, apart from subgenome A (*Fragaria vesca*), have been a subject of public controversy. Phylogenomic analyses revealed a close relationship between diploid species *Fragaria iinumae* and subgenomes B, C, and D. Subgenome A, closely related to *F. vesca*, retains the highest number of genes, exhibits the lowest content of transposable elements (TEs), experiences the strongest purifying selection, shows the lowest DNA methylation levels, and displays the highest expression level compared to the other three subgenomes. Transcriptome and DNA methylome analyses revealed that subgenome A-biased genes were enriched in fruit development biological processes. In contrast, although subgenomes B, C, and D contain equivalent amounts of repetitive sequences, they exhibit diverged methylation levels, particularly for TEs located near genes. Taken together, our findings provide valuable insights into the evolutionary patterns of subgenome structure, divergence and epigenetic dynamics in octoploid strawberries, which could be utilized in strawberry genetics and breeding research.

## Introduction

Cultivated strawberry (*Fragaria × ananassa* Duch, 8*n* = 56) is a widely grown hybrid species cultivated worldwide due to its significant economic value. The cultivated strawberry originated from the natural hybridization of *Fragaria virginiana* and *Fragaria chiloensis* in eighteenth-century Europe [1, 2]. Renowned for its delightful aroma, appealing fruit color, and juicy texture, the large-fruited strawberry is considered a superior alternative to *Fragaria vesca* and other wild species [3]. Over the years, farmers and breeders have developed new varieties with desirable traits suitable for various climates and flowering habits. Japan, in particular, has successfully produced its own cultivars, some of which have been introduced to other countries. ‘Benihoppe’ is an early maturing variety of cultivated strawberries derived from the crossbreeding of ‘Akihime’ and ‘Sachinoka’ in Shizuoka Prefecture, Japan [4]. Following its introduction to China, ‘Benihoppe’ has become one of the most widely cultivated varieties in the country. Its adaptability, shallow dormancy, large fruit size, and excellent quality have made it an ideal parental material and the subject of biochemical experiments. Statistic data of 105 strawberry varieties bred from 1953 to 2016 revealed that ‘Benihoppe’ was frequently used as a parent in hybridization breeding [5]. However, ‘Benihoppe’ is susceptible to powdery mildew and *Colletotrichum* Spp. pathogen, making it valuable for studying disease defense responses [6–8]. Researchers have conducted physiological studies on ‘Benihoppe’ to understand its responses to biotic and abiotic stresses [9–13], phenylpropanoid biosynthesis [14–16], and essential agronomic traits [17–20]. Additionally, ‘Benihoppe’ is an ideal receptor via *Agrobacterium*-mediated genetic transformation [21, 22].

To further advance fundamental and applied research, the availability of a fully phased reference genome of octoploid strawberry cv. ‘Benihoppe’ would greatly facilitate subgenome comparative studies. Polyploidy, or whole genome duplication (WGD), is a significant evolutionary event that can potentially enhance genetic breeding by influencing gene expression and phenotypic variation through genetic and epigenetic factors [23, 24]. When allopolyploid species arise from the fusion of two or more diploid species, each contributing one subgenome, they must rapidly resolve genetic incompatibilities between subgenomes. Incompatibilities might be caused by interactions between genes, transposable elements, and small RNAs that have functionally diverged in each species before polyploidization. The coordination and coexistence of subgenomes in the same nucleus can occur through two distinct pathways: dominant subgenome evolution or parallel subgenome structure evolution. The dominant subgenome evolution is characterized by the retention of more genes, increased expression of homoeologous, reduced repetitive sequences, and stronger selective pressure [25–28]. Examples of dominant subgenome evolution have been observed in various allopolyploid plants, such as Chinese cabbage (*Brassica rapa*), wheat (*Triticium aestivuum*), allo-tetraploid peanut (*Arachis hypogaea*), and cotton (*Gossypium hirsutum*) [29–32]. On the other hand, some polyploid species exhibit parallel subgenome evolution, characterized by similar repetitive sequence contents, high sequence synteny, and symmetric purifying selection of all subgenomes. Notable examples include allotetraploid common carp and goldfish [33].

DNA methylation plays a crucial role in maintaining the normal functions of cells, such as inactivating one X chromosome in female mammals, stabilizing genome structure, regulating embryonic development, and influencing the occurrence and development of diseases [34–36]. In plants, epigenetic variation, including DNA methylation, can induce changes in chromatin conformation, thereby regulating gene expression and transposable elements (TEs) silencing. Consequently, DNA methylation can contribute to species diversity and enhance environmental adaptability [37, 38]. Advances in sequencing technology have enabled the generation of haplotype-resolved genomes, facilitating the analysis of haplotype-resolved DNA methylation in various organisms, such as African *cassava* [39]. DNA methylation has been associated with TEs and heterochromatic intergenic regions in oaks [40]. In *Fragaria*, dynamic changes in DNA methylation have been linked to stress response, climate change adaptation, and dormancy induction in diploid species [41–45], and octoploid strawberries [46, 47]. However, previous studies on DNA methylation in octoploid strawberries have relied on diploid genomes due to the lack of high-quality octoploid assembly references at the subgenome level [41, 42, 46]. This has limited the study of subgenome-specific DNA methylation levels in octoploid strawberry.

In this study, we assembled a gap-free genome of the cultivated octoploid strawberry ‘Benihoppe’ using PacBio HiFi reads, oxford nanopore technology (ONT) ultra-long reads, and Hi-C technology, and analysed variations in DNA methylation among its subgenomes. Through multiple phylogenetic analyses of the four subgenomes of octoploid strawberry and available diploid genomes, we uncovered the ABBxBx genome structure of octoploid strawberry. Furthermore, we conducted a comparative analysis of subgenome structure, repetitive sequences, and DNA methylation, which revealed that subgenome A is dominant, while the other three subgenomes exhibit similar structures (parallel subgenome evolution). Transcriptome and DNA methylome analyses revealed subgenome A-biased genes were enriched in fruit development biological processes. Furthermore, the fruit mature-related genes were higher expressed in the A subgenome than in B/C/D subgenomes, which were largely gene body methylated. We anticipate that this high-quality assembly of octoploid strawberries will advance genetic studies in plant sciences and facilitate crop improvement and breeding.

## Results

### Gap-free genome assembly and annotation

*Fragaria × ananassa* cv. ‘Benihoppe’ was selected for genome sequencing and assembly. To develop a high-quality genome assembly, we generated ~64.5 Gb of HiFi subreads, 40 Gb of ONT ultra-long reads and 100 × high-throughput chromatin conformation capture (Hi-C) reads. Using the Hifiasm algorithm [48], we yielded two haplotigs of 851 Mb and 821 Mb, with contig N50 values of 25 Mb and 26 Mb, respectively. After scaffolding using the 3D *de novo* assembly pipeline (3D-DNA, [49]), 92.47% and 96.21% of contig sequences from the two haplotypes were anchored to 28 chromosomes (Fig. S1, see online supplementary material). We assembled the genome based on ONT ultra-long reads to close gaps. The final gap-free assembly consisted of 28 chromosomes that clustered into seven groups representing chromosomes one to seven. Chromosomes were identified from phylogenetic analysis using PhyDs [50]. Each block contained four subgenomes labeled as A, B, C, and D. The final genome assembly has a size of 793 Mb and exhibited 99.4% completeness in conserved single-copy protein-coding sequences based on the embryophyta_odb10 database in BUSCO. (Fig. S2, Table S1, see online supplementary material).

**Figure 1:**
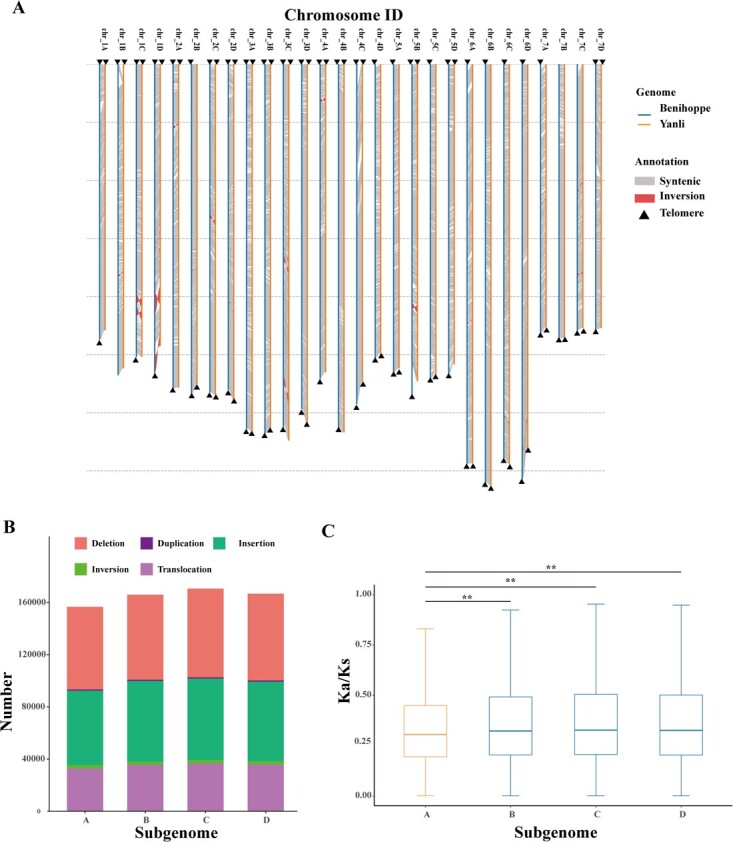
High-quality genome assembly and subgenome structure of ‘Benihoppe’. **A** Visualization of syntenic regions and structural rearrangements between ‘Benihoppe’ and ‘Yanli’ genomes. Triangles indicate telomeres. Grey and dark blue colors show chromosome syntenic and inversion regions in genomic sequences, respectively. **B** The number of different types of structural variants (SVs) in four subgenomes (deletions, duplications, insertions, inversions, and translocations). **C** Distribution of *Ka/Ks* ratios of protein-coding genes in each subgenome of ‘Benihoppe’. This difference was statistically significant using paired Wilcoxon signed rank tests (*P* value <0.01).

Using telomeric repeats (TTTAGGG)_n_ as a query [51], we identified 53 telomeres in 28 chromosomes of assembly (Fig. 1A). Plant centromeric regions usually comprise TEs and tandem repeats [52]. By utilizing Tandem Repeats Finder (TRF), we identified a cluster of 147 bp centromeric tandem repeats, ranging from kilobases (Kbs) to megabases (Mbs) in length, on 25 out of 28 chromosomes of ‘Benihoppe’ (Tables S2 and S3, see online supplementary material). The remaining three chromosomes 1B, 1C, and 1D carried continuous tandem repeats of 148 bp, 158 bp, and 145 bp, respectively (Fig. S3, see online supplementary material). To identify the location and sequence of centromeres in our gap-free genome, we used quarTeT [53] for centromere candidate prediction and got the similar results.

Visualizing the structural rearrangements between the entire assemblies of ‘Benihoppe’ and ‘Yanli’ [54] revealed a highly conserved synteny, except for small inversions located in chromosomes 1C, 1D, and 5B (Fig. 1A). The homologous chromosomes of the four subgenomes maintained a high level of collinearity, with some sequence rearrangements (Fig. S4A, see online supplementary material). Notably, a structural variant was observed at the end of homologous chromosome 1, which was confirmed by a Hi-C matrix (Fig. S4B, see online supplementary material). Additionally, a large inversion and translocation were detected on chr_2C, consistent with recent studies on chromosome 2–1 of ‘Yanli’ assemblies (Fig. S4B, see online supplementary material) [54]. Inversions and translocations were found to occur frequently between chromosomes 3 and 6 and between chromosomes 3 and 7 (Fig. S4A, see online supplementary material). For example, collinearity analysis of subgenome A revealed sequence rearrangements between chromosomes 3A and 6A, involving 248 protein-coding genes (PCGs) associated with Gene Ontology (GO) terms of ubiquitin-protein transporter activity and positive regulation of metabolic processes (Fig. S4C, see online supplementary material).

Structural variants (SVs), including deletion, duplication, insertion, inversion, and translocation that contribute substantially to phenotypes and plant domestication [55, 56], were identified across subgenomes using PacBio raw data. Subgenomes A and C exhibited the minimum and maximum number of SVs (i.e., 156 684 and 170 564, respectively) (Fig. 1B; Table S4, see online supplementary material), suggesting that the chromosome structure of subgenome A is more stable than the other subgenomes. To assess the selection pressure acting on the four subgenomes, we calculated both the nonsynonymous substitution rate (Ka) and the synonymous substitution rate (Ks) based on homoeologous gene pairs. The median *Ka*/*Ks* values in subgenome A is significantly lower than other three subgenomes (B/C/D), indicating stronger purifying selection in subgenome A (*P* value <0.01, Wilcoxon test) (Fig. 1C). It is well known that the dominant subgenome experienced stronger purifying selection pressure and exhibited higher homeologous gene expression in many allopolyploid genomes of plants and animals [57, 58]. Our observation shows that subgenome A is dominant as its genes evolved under greater purifying selection.

**Figure 2:**
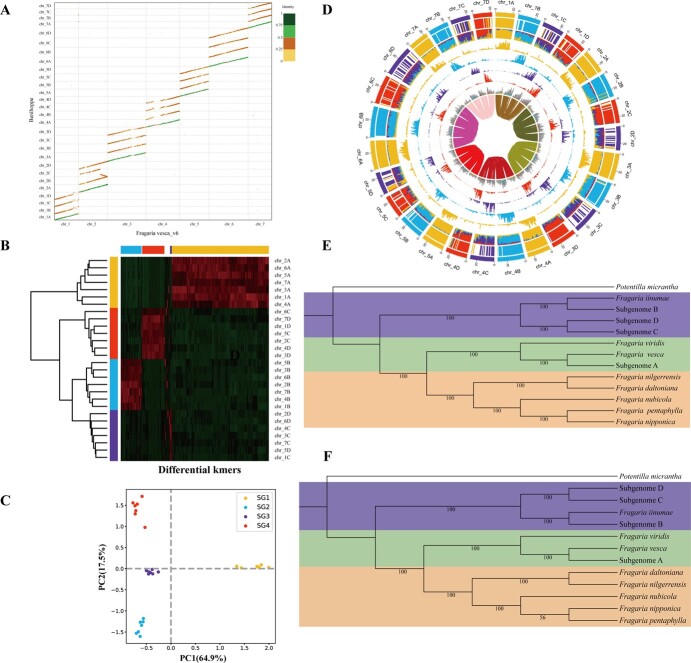
Subgenome phasing of ‘Benihoppe’ assembly. **A** Dot plot of ‘Benihoppe’ assembly and *Fragaria vesca* v6.0 genome. The evolutionary origin of the ‘Benihoppe’ subgenomes is identified by PhyDS. **B**, **C**, and **D** Subgenome phasing and characterization of ‘Benihoppe’ using SubPhaser. **B** The heatmap indicates the abundance of 15-mers. **C** Principal component analysis (PCA) of differential 15-mers. **D** Circos plot of chromosomal characteristics. From outer to inner circles (1–9): (1) Subgenome assignments based on k-means algorithm; (2) Significant enrichment of subgenome-specific k-mers – the same color as the subgenome indicates significant enrichment for those subgenome-specific k-mers; white areas are not significantly enriched; (3) Normalized proportion (relative) of subgenome-specific k-mers; (4–7) count (absolute) of each subgenome-specific k-mer set; (8) Density of long terminal repeat retrotransposons (LTR-RTs) – if the color is consistent with the subgenome, it indicates that LTR-RTs are significantly enriched to those subgenome-specific k-mers; gray indicates nonspecific LTR-RTs; (9) homoeologous blocks. All statistics (2–8) are computed in sliding windows of 1 Mb. **E** The maximum likelihood (ML) phylogenetic tree among the ‘Benihoppe’ subgenomes and diploid genomes, inferred by IQ-TREE on concatenated genes with 1000 bootstraps. **F** The phylogenetic tree based on coalescence using ASTRAL. With Potentilla micrantha genome as the outgroup.

For gene annotation, the mRNA data were aligned to the assembly of ‘Benihoppe’, resulting in the prediction of 109 320 protein-coding genes, with 96.0% complete BUSCOs (Fig. S2, Table S1, see online supplementary material). These results highlighted the high accuracy and quality of our genome assembly. We annotated 26 260–29 597 genes for each subgenome, with subgenome A containing the highest number of genes (Table S5, see online supplementary material).

Our assembly and annotation provided support for a gap-free octoploid strawberry genome, which can be valuable for studying genes and breeding. The analysis of the subgenome structure revealed that subgenome A is dominant, as it has evolved under greater purifying selection and retained the highest number of genes.

### Phasing the subgenomes of ‘Benihoppe’ assembly and inferencing the phylogenetic trees

For octoploid strawberry, subgenome-level analysis is greatly hindered for its diploid ancestors are unknown or extinct. Based on the phylogenetic analysis of nuclear genes in the ‘Camarosa’ genome, we named the 28 chromosomes chr_1A to chr_7D in our assembly. Alignments of ‘Benihoppe’ assembly against the latest diploid genome of *F. vesca* v6.0 [59] showed that subgenome A, including chromosome 1A – 7A, shared a remarkably high degree of identity to *F. vesca* (Fig. 2A). To verify the chromosome phasing result, we phased the subgenomes of ‘Benihoppe’ assembly using SubPhaser v1.2 [60]. We scanned 15-bp sequences (15-mers) in all chromosomes and identified chromosome-specific 15-mers. Four distinct groups are labeled as SG1, SG2, SG3, and SG4 (Fig. 2B–D). Clustering and principal component analysis (PCA) of the k-mers showed PC1 clearly separate SG1 from SG2, 3, and 4. PC2 does not show mixture between SG2, 3, and 4 but a progressive segregation between 2 and 3 and 3 and 4 (Fig. 2C). Our phasing results based on k-mer are therefore not exactly the same as our assembled subgenomes C and D.

To test whether the subgenome phasing error was confounding our inferred evolution of cultivated strawberry, we conducted maximum likelihood (ML) phylogenetic tree (Fig. S5A, see online supplementary material) and coalescence-based tree (Fig. S5B, see online supplementary material) among subgenomes and candidate ancestral diploid donors. The results suggested that subgenomes B, C, and D originated from the same ancestor (i.e., an unsampled or extinct population of *F*. *iinumae*). We also conducted a maximum likelihood phylogeny separately for each chromosome 1 to 7 with IQ-TREE. The results lead to a similar conclusion where species tree got. Subgenomes C and D are commonly sister groups except for the tree inferred from single-copy genes of chromosome 5 (*n* = 808) and chromosome 7 (*n* = 594) (Fig. S6, see online supplementary material). This may indicate that subgenomes C and D originated from closely related yet unknown ancestors. The close relatedness of subgenomes C and D likely explains the potential errors in the phasing of these two subgenomes.

To gain insight into the genetic diversity and evolution of *Fragaria* species, we then inferred species/subgenome trees using ML and coalescence-based methods to draw phylogenetic trees. The ML phylogenetic tree revealed two distinct clades. Clade one comprised *Fragaria iinumae* and subgenomes B, C, and D, while clade two comprised *Fragaria viridis*, *F. vesca*, subgenome A and other diploid genomes (*Fragaria daltoniana*, *Fragaria nilgerrensis*, *Fragaria nubicola*, *Fragaria nipponica*, *Fragaria pentaphylla*) (Fig. 2E). The coalescence-based tree was consistent with the ML species tree (Fig. 2F). The tree inferred from the whole chloroplast genomes of 21 *Fragaria* species further suggested a close evolutionary relationship between subgenome A and *F. vesca* and between subgenome B and *F*. *iinumae* [61]. These results support the conclusion drawn by Edger *et al.* [50] and Liston *et al.* [62] regarding the progenitors of subgenomes A and B. Our results provide that *F*. *iinumae* is the progenitor of subgenome B. The phylogenetic analysis also indicated that subgenomes C and D were closely related to *F*. *iinumae*. These evolutionary origins of octoploid strawberry subgenomes suggest that *F. vesca*, *F. iinumae*, and two *F. iinumae*-like ancestors have contributed to the evolution of allo-octoploid cultivated strawberries. These findings provided additional support for the ‘Benihoppe’ genome originating from four diploid ancestors named ABBxBx.

### Evolutionary analysis of long terminal repeat retrotransposon families in the ‘Benihoppe’ assembly

**Figure 3:**
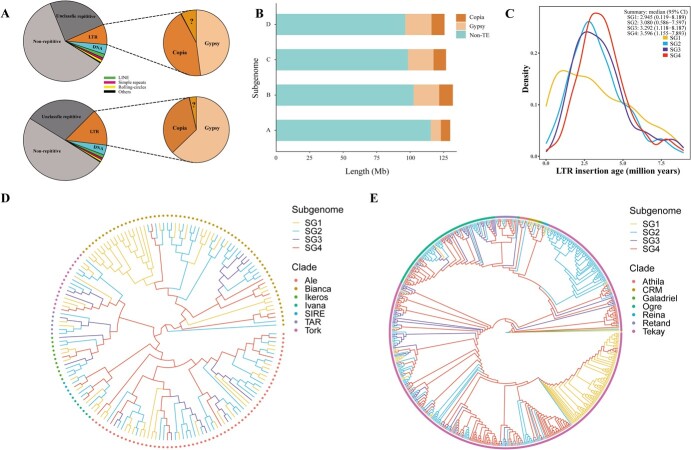
Identification of repetitive sequences and evolutionary analysis of long terminal repeat retrotransposon (LTR-RT) families in subgenomes of the cultivated strawberry genome. **A** The portion of repetitive sequences of subgenome A (upper) and others (i.e. subgenome B, C, and D, lower). **B** The sequence length of Copia and Gypsy in four subgenomes. **C** Insertion times of subgenome-specific LTR-RTs. The 95% confidence interval (CI) is marked to infer the insertion time boundary of LTR-RTs on the subgenomes. **D** and **E** Phylogenetic tree of Copia (**D**) and Gypsy (**E**). The branches are colored by subgenomes and terminal nodes are colored by clades.

Repetitive sequences, including TEs, have been recognized as a significant contributor to genome size variation and gene expression diversity. Similar to many plant species, the ‘Benihoppe’ subgenomes exhibit various levels of repetitive sequences, as determined by RepeatMasker analysis using the *Fragaria* library constructed by RepeatModeler. In subgenome A, a total of 40.02% of sequences were identified as repetitive sequences, including 8.14% of long terminal repeat retrotransposon (LTR-RT), 3.91% of DNA transposons, 1.57% of long interspersed nuclear elements (LINEs), and 0.95% of simple repeats (Fig. 3A and B; Table S6, see online supplementary material). Comparatively, the latest *F. vesca* v6.0 genome assembly [59], known as the origin of subgenome A, exhibited a lower proportion of repetitive elements (35.63%), suggesting that subgenome A accumulated TEs after polyploidization. Subgenomes B, C, and D contained ~50% repetitive sequences, with a similar distribution of each repetitive type except for LTRs (Fig. 3A and B; Table S6, see online supplementary material). LTR-RT, specifically the Copia and Gypsy classes, represented the majority of identified repetitive sequences. In subgenome A, about 3.6% of Copia and 3.9% of Gypsy elements were identified, while other subgenomes displayed a comparable proportion of Copia, with 4.78%–5.02%, but a very different proportion of Gypsy 9.26%–10.10% (Table S6, see online supplementary material). This suggests that subgenomes B, C, and D are parallel subgenomes. They have evolved with similar levels of transposons.

Previously studies have reported that genes in proximity to TEs are more susceptible to methylation and silencing [63]. We computed the distance between each gene and its closest TE in all subgenomes. Subgenome B exhibited the highest proportion of genes within 500 bp of the nearest TEs, with about 74.12% of genes falling into this category (Table S7, see online supplementary material).

Repetitive sequences, including satellite tandem repeats and TEs, often exhibit a preferential distribution within the genome [64]. Among these repetitive sequences, transposons are frequently found in telomeres and centromeres [52, 65]. Fig. S7 (see online supplementary material) illustrates the genome-wide distribution of genes, TEs, and centromeric regions. A negative correlation was observed between the distributions of genes and centromeres. Genes were depleted or absent around heterochromatic regions, including telomeres and centromeres. The centromeric regions of most chromosomes were predominantly colonized by Gypsy and Copia retrotransposons (Fig. S7, see online supplementary material), consistent with the distribution of TEs observed in oaks [40]. In *Helianthus* species, Ty3/gypsy-like sequences and Ty1/copia-like sequences tended to be present at the centromeric regions and chromosome ends, respectively [66].

We inferred the time boundary of subgenomes divergence to the hybridization period by estimating the subgenome-specific LTR-RTs insertion time (Fig. 3C). The LTR-RTs insertion time of SG3 and SG4 were similar, ranging from ~8.1 to ~1.1 million years ago (MYA) (95% confidence intervals). The estimated time of SG1 was distinct from other subgenomes (ranging from ~8.1 to ~0.1 MYA). These results suggested that SG3 and SG4 were more likely to have been introduced into the cultivated strawberry genome at the same time and SG1 was the last donor of octoploid strawberry. We also inferred the phylogenetic tree of Copia and Gypsy (Fig. 3D and E). It seems that the TE phylogeny recapitulates the diploid species divergence as many TE groups are subgenome-specific. The TE phylogeny also shows the effect of the polyploidization event through terminal branches showing recent introductions of TEs into new subgenomes after polyploidization. These results shed light on the subgenomes involved in the origin of octoploid strawberry.

### Dynamics of gap-free DNA methylation of subgenomes

**Figure 4:**
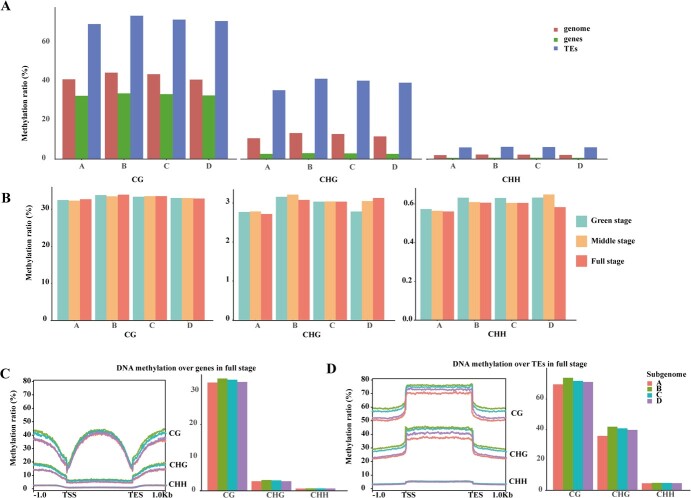
Patterns of DNA methylation over the entire genome, genes, and TEs during fruit ripening. **A** The average weighted methylation level for genome, genes, and TEs in the three contexts: CG, CHG, and CHH. **B** Average methylation levels in genes at three fruit development stages (green stage, middle stage, and full stage) in CG, CHG, and CHH methylation contexts. **C** and **D** Global distribution of DNA methylation levels at genes and TEs, respectively, including a 1-kb window upstream of the TSS and downstream of the transcription end site (TES).

To explore the dynamics of DNA methylation in the gap-free subgenomes of octoploid strawberries, we utilized high-quality octoploid assemblies and whole-genome bisulfite sequencing (WGBS) data, which enabled fine-scale resolution of 5-methylcytosine (5-mC) methylation in octoploid strawberry subgenomes. Specifically, we investigated DNA methylation patterns during fruit ripening at the green, middle, and full stages using bisulfite-seq data from a previous publication [46]. Our analysis revealed that developing strawberry fruits displayed a high average CG methylation level over TEs (71.84%) compared to the genome-wide methylation levels (42.52%) and gene methylation levels (33.16%) on average (Fig. 4A). Similar patterns were observed in the CHG and CHH contexts, consistent with previous findings in plants, indicating high methylation levels in TEs [67]. Next, we examined the DNA methylation levels over protein-coding genes during fruit ripening. Methylation levels in CG context over genes varied across subgenomes and fruit ripening stages (Fig. 4B). Subgenomes A and B exhibited the lowest CG methylation level in the middle stage and the highest CG methylation level in the full stage (Fig. 4B). In contrast, subgenome D exhibited the highest CG methylation level in the green stage and the lowest CG methylation level in the full stage (Fig. 4B). When considering the average CG methylation level across all four subgenomes, the lowest methylation level was observed in the middle stage and the highest in the full stage, with 33.16% at the green stage, 33.08% at the middle stage, and 33.25% at the full stage. In contrast to the CG context, methylation level in the CHG context surrounding genes increased in subgenome D during fruit maturation (Fig. 4B), while the average CHG methylation level declined in subgenomes A, B, and C during fruit maturation (Fig. 4B). Regarding the CHH context, the lowest methylation level was detected in the full stage for subgenomes A, B, C, and D (Fig. 4B). Notably, our findings, based on the octoploid strawberry genome assembly, provided more precise insights into the methylation dynamics of subgenomes during fruit ripening compared to a previous study using diploid strawberry genome reference [46]. Overall, ripe fruits displayed higher CG methylation levels and lower CHG and CHH levels than immature fruits over genes.

We investigated the DNA methylomes in each subgenome, focusing on methylation patterns in genes and TEs. In general, subgenome A exhibited lower methylation levels in all contexts compared to the other subgenomes, particularly in the regions of TEs. In contrast, despite a similar proportion of repetitive sequences in subgenomes B, C, and D, the overall methylation levels at TEs were the highest in subgenome B in ripe fruits (Fig. 4C and D). These patterns were consistently observed during fruit development and maturation in octoploid strawberries (Fig. 4C and D; Fig. S8, see online supplementary material).

The divergence of DNA methylation among subgenomes B, C, and D may be attributed to the location of TEs, as subgenome B exhibited a shorter distance between genes and the nearest TEs (Table S7, see online supplementary material). Notably, the presence of intragenic TEs, which were inserted within gene bodies, could potentially reduce CHG methylation during the evolution of allotetraploid cotton [32]. Furthermore, intragenic TEs displayed lower levels of DNA methylation than intergenic TEs in both CG and CHG contexts (Fig. S9, see online supplementary material). This disparity between intergenic and intragenic TEs may be a result of selection pressure favoring gene expression, as observed in previous findings [68]. Additionally, CG and CHG methylation levels of intragenic TEs were reduced to a lower level in subgenome D than in subgenome B. Despite the presence of similar TEs, subgenome B exhibited higher DNA methylation levels than subgenomes C and D (Fig. 4D).

### Gene expression changes among subgenomes of ‘Benihoppe’ assembly

To investigate the gene expression changes among the four subgenomes, we retrieved two biological replicates of fruit RNA-seq samples at green, middle and full stage, respectively. As subgenome A was the dominant one, we focused on the comparison of homeolog gene pairs between subgenome A and the other three subgenomes, i.e., B/C/D. On average, we detected 21 328 homeologs gene pairs between subgenome A and the other three subgenomes. Then we estimated their expression changes among subgenomes using two computation methods.

**Figure 5:**
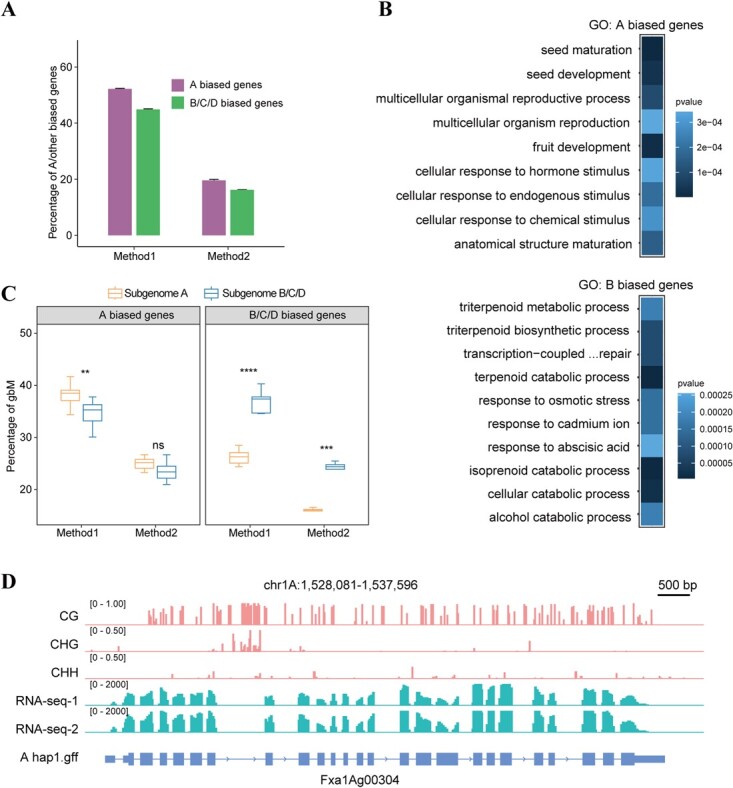
Expression and gene body methylated (gbM) among subgenomes of ‘Benihoppe’ assembly. **A** The percentage of homeologous gene pairs among four subgenomes with different degrees of expression bias. **B** GO annotations of subgenome A-biased and B-biased protein-coding genes present in the ‘Benihoppe’ assembly. **C** The percentage of gbM in A-biased genes and B/C/D-biased genes. Method 1 and method 2 were described in the section ‘Inferences of gene body methylation’. **D** IGV tracks show methylation and RNA-seq levels of *F×a1Ag00304*, a A-biased gbM gene. The CG, CHG, and CHH patterns are shown in the *F×a1Ag00304* gene. Method1: Estimation of A and B/C/D biased genes based on the different transcript numbers between homeologous gene pairs; Method2: Estimation of A and B/C/D biased genes by performing edgeR algorithm for homeologs gene pairs.

Firstly, we compared the transcripts numbers between homeologs gene pairs. On average, a median of 11 135 homeologs gene pairs (52%) was expressed higher in subgenome A than B/C/D. These genes are hereafter referred to as A-biased genes. On the other hand, a median of 9568 homeologs gene pairs (45%) was expressed at a higher level in subgenome B/C/D than in subgenome A, i.e., B/C/D-biased genes (Fig. 5A; Table S8, see online supplementary material). The results indicated the proportion of A-biased genes was higher than that of B/C/D-biased genes. Then, we investigated the biological roles of A-biased genes and B/C/D-biased genes based on the top 1000 expressed genes. Gene ontology (GO) analysis of these A-biased genes was enriched in biological processes such as seed maturation and development, multicellular organism reproduction, fruit development, cellular response to hormone/endogenous/chemical stimulus, and anatomical structure maturation (Fig. 5B; Tables S10 and S11, see online supplementary material). In contrast, GO analysis of the B/C/D-biased genes was enriched in biological processes such as triterpenoid metabolic/biosynthetic process, response to osmotic stress, and response to cadmium ion (Fig. 5B; Table S9, see online supplementary material).

Secondly, we employed edgeR [69] to define A-biased genes and B/C/D-biased genes. Like the first method, we detected a higher proportion of A-biased genes relative to B/C/D-biased genes. On average, a median of 4208 homeolog gene pairs (19.7%) were A-biased genes, while 3451 were B/C/D-biased genes (16%) (Fig. 5A;Table S10, see online supplementary material). The remaining genes exhibited no significant difference in expression level between subgenomes. We also performed GO analysis for the A- and B/C/D-biased genes, respectively. GO analysis of A-biased genes were enriched in biological processes such as seed maturation and pollen tube reception, and GO analysis of B/C/D-biased genes were enriched in the biological process such as cellular hyperosmotic response, terpenoid catabolic process, cellular response to water stimulus (Table S11, see online supplementary material). Consistent with the previous report [50], our study suggested that the dominant subgenome A had a higher expression, and the expression changes between dominant subgenome A and subgenome B/C/D largely contributed to key traits during strawberry fruit ripening.

### Correlation between DNA methylation and gene expression

To query the relationship between gene expression changes and the methylation status of individuals, we applied a statistical approach to determine whether genes were methylated above or below the genic background level for each subgenome (see Materials and Methods). Given their important role in fruit maturity, we focused on the analysis of the top 1000 A-biased and B/C/D-biased genes based on method1 (the transcript number comparison), and method2 (A-biased and B/C/D-biased genes defined by edgeR, see Materials and Methods). Across the three fruit maturation stages, the proportion of gene body methylated genes (gbM) in subgenome A were higher than the proportion of gbM in subgenome B/C/D for A-biased homeologs gene pairs. For example, for the top 1000 A-biased genes (based on method1), a median of 385 (38.5%) and 353 (35.3%) gbM were found in subgenome A and B/C/D subgenome (Fig. 5C;Tables S12 and S13, see online supplementary material), respectively. Similarly, in method2, for the 4232 A-biased genes, we detected a median of 1055 (25.2%) and 998 (23.4%) gbM in genome A and in B/C/D subgenomes, respectively (Fig. 5C; Tables S14 and S15, see online supplementary material). These results were consistent with previous studies showing that gbM was positively correlated with gene expression [3, 70]. For example, the gene *F×a1Ag00304* shown in Fig. 5D was an A-biased gene, gene body-methylated and related to fruit development. The pattern that gbM was associated with increased gene expression was further validated by the B/C/D-biased homeolog gene pairs, as we detected a higher proportion of gbM in B/C/D subgenomes than A subgenome, i.e., 263 (26.3% of 1000 genes) gbM in A subgenome vs. 374 (37.4% of 1000 genes) in B/C/D subgenome in the median, and 552 (16.1% of 3447 genes) in genome A vs. 841 (24.4% of 3447) in B/C/D subgenomes. The results suggested the mature fruit mature-related genes were more highly expressed in A subgenome than in B/C/D subgenomes, and were largely gene body methylated.

## Discussion

In this study, we successfully assembled a high-quality, gap-free genome of the cultivated strawberry (*Fragaria × ananassa*) variety ‘Benihoppe’. ‘Benihoppe’ is widely used in Chinese traditional cross-breeding to develop various strawberry varieties due to its early maturity, palatable taste, and excellent agronomic characteristics. However, the susceptibility of ‘Benihoppe’ to diseases such as powdery mildew and anthracnose caused by *Colletotrichum* Spp. limits its potential for large-scale production and scientific research applications. To address this limitation, we aimed to assemble the high-quality genome of ‘Benihoppe’ to facilitate gene identification and breeding improvement.

Previous studies have shown that the application of salicylic acid-primed defense response in ‘Benihoppe’ leaves can enhance its resistance against *Podosphaera aphanis,* the causal agent of powdery mildew [6]. Furthermore, comparative transcriptomic analysis of ‘Benihoppe’ has provided valuable insights into the molecular mechanisms underlying resistance against C. *gloeosporioides* in octoploid strawberries [8, 11]. Despite its weak disease resistance, ‘Benihoppe’ remains an almost ideal strawberry variety. Therefore, we aimed to assemble its high-quality genome to facilitate gene identification and breeding strategies to improve disease resistance in cultivated strawberries.

Initially, the lack of a well-assembled genome for the octoploid strawberries necessitated the use of the *F. vesca* genome as a reference in the analysis of *F*. *ananassa* before the publication of the first genome of octoploid ‘Camarosa’ cultivar [50]. The chromosome-level assembly of the octoploid strawberry ‘Camarosa’ has significantly improved the identification of novel genes associated with specific traits and facilitated molecular marker-assisted breeding in strawberries [50, 71, 72]. This assembly also allowed the identification of the four diploid progenitors for the first time. However, the Camarosa v1 genome assembly still requires improvements to address local phasing errors and fragmented assembly of subgenomes.

Assembling the allo-octoploid strawberry genome has been challenging due to its high heterozygosity and complexity. A highly homozygous inbred line of ‘Benihoppe’ named ‘Wongyo 3115’ was utilized to generate a chromosome-level assembly, which facilitated the construction of a high-density genetic map and the identification of quantitative trait locus (QTL) for fruit firmness gene mapping [73]. The availability of high-quality genomes is crucial for in-depth analysis, and HiFi reads, with their long-read and high accuracy, have been employed for the assembly of complex and large genomes [74–78]. Telomere-to-telomere genomes have been generated using HiC or/and ONT data, further enhancing genome assembly [51, 79–82]. Utilizing the trio-binning assembly strategy, third-generation HiFi sequences and Illumina paired-end sequences of parent genotypes has allowed us fully phase the genome of the American day-neutral strawberry cultivar ‘Royal Royce’ [83]. Additionally, a high-quality haplotype-resolved genome of the octoploid strawberry ‘Yanli’ revealed structural diversity and complexity in the expression of genes in the anthocyanin biosynthesis pathway [54] (Table S16, see online supplementary material). We compared the length of corresponding chromosome sequences between the genomes of ‘Benihoppe’ and ‘FaRR1’, ‘Yanli’, which were assembled by HiFi reads (Fig. S10, Table S17, see online supplementary material). These high-quality genomes are instrumental in discovering genomic variations and constructing pan-genomes.

Strawberry, unlike certain plants like wheat (*T. aestivuum*), allotetraploid peanut (*A. hypogaea*), cotton (*G. hirsutum*), and other allo-polyploid plants, lacks a standardized nomenclature for chromosomes and subgenomes [29, 31, 32]. Based on an independent linkage group, nomenclatures with differing subgenome compositions have been applied to strawberries [83]. Unknown diploid ancestors of octoploid strawberries make it hard to phase subgenomes and unravel the subgenomes and genome evolution directly. In our study, we adopted chromosome names that represent the original diploid ancestors of subgenomes A, B, C, and D by PhyDs. We also phased the subgenomes by searching for the subgenome-specific sequence (k-mer) using SubPhaser [60]. Comparing the phasing results, subgenomes A and B were in accordance with SG1 and SG2. Subgenomes C and D were different from SG3 and SG4 in chromosomes 2, 5, and 6. We then inferred the ML phylogenetic tree among subgenomes and diploid species of chromosome 1–7. We concluded the similar subgenome evolution relationship. It is most likely that SG3 and SG4 are commonly placed as sisters to each other. In short, we applied subgenomes A, B, C, and D for chromosome nomenclature and the adoption of a uniform chromosome nomenclature facilitates the integration of genetic and physical mapping data.

Determining the diploid progenitors contributing to cultivated strawberries has been challenging. Phylogeny and genomic investigations have been carried out to shed light on this matter. Previous studies support *F. vesca* and *F*. *iinumae* as two diploid ancestors [50, 62, 84, 85, 86]. However, the identity of the remaining progenitors remains unknown. Different hypotheses propose two *F*. *iinumae*-like ancestors [84]: *F*. *bucharica*, *F. viridis*, and an unknown diploid [85], or *F. viridis* and *F*. *nipponica* [50]. Leveraging the high-quality ‘Benihoppe’ assembly, we analysed evolutionary relationships and sequence collinearity of the four subgenomes and diploid genomes. First, we phased the subgenomes in two methods as mentioned above. In addition, we inferred the ML phylogenic trees based on chromosome sing-copy genes. Species trees were inferred by IQ-TREE and coalescence-based methods (Fig. 2E and F). Our comprehensive set of analyses strongly supports an ABBxBx subgenome structure of cultivated octoploid strawberries, with subgenome A originating from *F. vesca*, subgenome B from *F*. *iinumae*, and subgenomes C and D from species closely related to *F*. *iinumae*. Nevertheless, the telomere-to-telomere genome of *F. vesca* reveals an AA.AA.AA.BB model of octoploid strawberry, with AA and BB representing *F. vesca* and *F. viridis*, respectively [59]. Zhou *et al.* divided the genome of *F. × ananassa* FL15.89–25 into four subgenomes and inferred the contrary phylogenomic result. However, Zhou *et al.* did not say how they divided the octoploid genome into subgenomes. Without such information, we could not compare our analysis to theirs. Furthermore, we performed genome syntenic relationships between ‘Benihoppe’ assembly and *F.iinumae, F. viridis*, visualizing regions with sequence similarity greater than 90%. Subgenome B exhibited higher levels of collinearity with *F. iinumae* (Fig. S11, see online supplementary material). Additionally, the haplotype network of chloroplast genomes from 21 *Fragaria* species revealed that *F. vesca* subsp. bracteata was the most recent maternal donor of the octoploid strawberry [61]. Our LTR-RT insertion time supported that SG3 and SG4 were likely to have been introduced into the octoploid strawberry genome at the same time. Therefore, we insisted that tetraploid and hexaploid species may have played an evolutionary intermediate role between diploid and octoploid species.

TEs usually are known to constitute a significant proportion of eukaryotic genomes, ranging from 10% in *Arabidopsis thaliana* to 85% in *Zea mays* [87, 88]. In octoploid strawberry genomes, TE-related sequences make up ~36% to 45.05% of the total content. Specifically, in the ‘Benihoppe’ assembly, repetitive sequences account for 40.02% in subgenome A and ~50% in other subgenomes (Fig. 3A). Among TEs, LTR retrotransposons, particularly Copia and Gypsy retrotransposons, are the most abundant in the strawberry genome. The discrepancy in the abundance of Copia and Gypsy retrotransposons of subgenome A and other subgenomes is a notable feature. The expansion of TEs, especially LTR-RTs, contributes significantly to genome size. For instance, the genome size of diploid cotton *Gossypium arboretum* (2×) is twice that of *Gossypium raimondii* (2×), with TEs accounting for 57% and 68.5% of their genomes, respectively [89]. The lower proportion of TEs in subgenome A may explain its relatively small size compared to other subgenomes. It is worth noting that in many polyploid genomes, the subgenome with the fewest gene losses often exhibits the lowest TE density [50].

Genic methylation tends to occur in the CG context within transcribed regions, and this gene body methylation (gbM) is linked to active transcription [90–94]. GbM genes are typically transcribed at moderate to high levels and across more tissues relative to unmethylated genes (e.g. [93, 94]). The dominant subgenomes in *Z. mays* [28, 95], *B. rapa* [30], *Gossypium* [96], and *Fragaria ananassa* [50] have been observed to possess lower TE abundance. Consistent with this pattern, our analysis revealed that subgenome A is dominant in the octoploid strawberry, characterized by a higher number of PCGs, lower repetitive sequence contents, and stronger selective pressure. The lower TE content in subgenome A also correlates with lower DNA methylation levels across both genes and TEs in all sequence contexts. In contrast, subgenomes B, C, and D showed parallel evolution, with a similar number of retained genes and comparable TE content. Despite the presence of equivalent repetitive sequences in subgenomes B, C, and D, DNA methylation levels differ (Fig. 4; Fig. S9, see online supplementary material). We speculate that the genome structure variation caused by TEs can play a crucial role in shaping plant epigenomics, particularly through the influence of TE distance to genes on DNA methylation patterns. For instance, we observed highest DNA methylation levels at TEs in subgenome B, which could be attributed to the shorter distance between genes and the nearest TE in that subgenome.

## Conclusion

In conclusion, our gap-free assembly of the octoploid strawberry genome provided insights into subgenome-level variation and genome structure. Our findings support an ABBxBx subgenome structure for octoploid strawberry, with subgenome A being dominant and the other subgenomes exhibiting parallel evolution patterns, possibly as a consequence of their close relationship to the *F*. *iinumae* common ancestor. The distinct DNA methylation patterns among subgenomes B, C, and D suggest that intragenic TEs and intergenic TEs may drive epigenetic changes in genes. The dominant subgenome A had higher expression genes that largely contributed to key traits during strawberry fruit ripening. And the mature fruit mature-related genes were largely gene body methylated in subgenome A. These findings highlight the complex evolutionary and origin of allopolyploid genomes.

## Materials and methods

### Genome sampling

*Fragaria × ananassa* Duch ‘Benihoppe’ is one of the widely grown cultivars worldwide. Fresh and tender leaves were taken from the ‘Benihoppe’ plants at the strawberry greenhouse of Zhengzhou Fruit Research Institute, Chinese Academy of Agricultural Sciences.

### DNA extraction, library construction, and sequencing

High-quality genomic DNA was extracted from the gender leaves of *F*. *ananassa* cv. ‘Benihoppe’, following the protocol of a modified cetyltrimethyl ammonium bromide (CTAB) method [97]. The SMRT cell sequencing library containing about 15–20 kb DNA fragments was constructed and sequenced using PacBio sequel II platform. A Hi-C library was established and sequenced by the Illumina NovaSeq 6000 platform (Illumina, San Diego, CA, USA). A PromethION was used for ultra-long ONT sequencing (Oxford Nanopore Technologies, Oxford, UK). De novo genome assembly.

A total of ~64.5 Gb of HiFi reads with ~80× coverage was generated. The average and N50 of the circular consensus sequencing (CCS) reads are 14 231 bp and 14 215 bp, respectively. A Hi-C library was constructed by the gender leaves of the ‘Benihoppe’ strawberry plant. A total of ~282 million (~100×) 150 bp paired-end (PE) sequences were produced on the Illumina NovoSeq 6000 platform. The Illumina reads were cleaned using fastp (v.0.23.1) [98] software with the default settings. Hifiasm (v.0.16.1-r375) [48] was used to generate haplotype-resolved assemblies using HiFi and paired-end Hi-C reads with the default command. In this mode, a fully phased contig graph of haplotype1 and haplotype2 was generated. The size of haplotigs (a contig of clones with the same haplotype) is ~851 Mb and ~ 821 Mb. Clean Hi-C reads were mapped to the haplotigs separately to produce a Hi-C contact matrix with Juicer (v.1.6). We subsequently used 3D-DNA pipeline [49] to sort and orientate contigs, generating 28 pesudochromosomes in each haplotype. The pesudochromosomes were manually adjusted by JuiceBox (v.1.11.08). Last, we assembled two haplotype genomes after conducting run-asm-pipeline-post-review.sh script with 3D-DNA. We adjusted the assemblies manually through the HiFi and ultra-long ONT reads, which were mapped to the final haplotypes with minimap2 (v.2.21). Telomere-to-telomere genome assembly methods referred to Shi *et al.* [99]. Benchmarking Universal Single-Copy Orthologs (BUSCO) [100] was used to evaluate the completeness of genomes with the Embryophyta_odb 10 database.

### Annotation of repetitive sequence

RepeatMasker (v.4.1.2) was utilized to mask the genome and annotate repetitive sequences in the forms of tandem repeats and interspersed repeats in both haplotype genomes. The repeat sequence libraries were constructed with the genomes of *F. vesca* Hawaii-4 [101] and *F. ananassa* cultivar ‘Camarosa’ [50] by RepeatModeler (v.2.0.1), which can be applied with RepeatMasker.

### RNA sequencing and genome annotation

Tissues of roots, leaves, flowers, and different stages of developing fruits were liquid nitrogen freezing and stored at −80°C. Total RNA was isolated by standard TRIzol protocol. Protein coding genes of ‘Benihoppe’ was annotated using MAKER2 pipeline (v.3.01.03 [102]) by *de novo* prediction, homology-based gene prediction, and transcriptome-based gene prediction. First of all, the cleaned RNA sequence data was mapped to our genome assembly using HISAT (v.2.2.1). The resulting genomic sequences were used to train the gene model by SNAP (v.2006-07-28). After two rounds of SNAP training, we obtained a mature gene model by the HMM algorithm. AUGUSTUS (v.3.4.0) was used to perform ab initio gene prediction. The third round of gene training was used by AUGUSTUS. The final predicted genes were verified and corrected by PASA.

### The identification of telomeres and centromeres

Tidk (v.0.2.0) (https://github.com/tolkit/telomeric-identifier) was used to identify and visualize telomeric repeats in the genome. The tidk explore was used to find the telomeric repeat unit in the genome. (TTTAGGG)n was the conserved telomeric repeats identified in most plants in the Telomere Database (http://telomerase.asu.edu/sequences_telomere.html). The tidk search was to identify the repeat sequence across the genome. Finally, we plotted the CSV output to visualize the telomere peaks. The centromere region is featured with a high density of short tandem repeats. The TRF software (v.4.09) [103] was used to identify tandem repeats. Then we merged the outputs by using TRF2GFF (https://github.com/Adamtaranto/TRF2GFF). We analysis the statistics in IGV (v.2.12.3) [104]. We used characteristics to identify continuous clusters of each chromosome.

### Identification of homologous genes under selection

The homologous genes of *Potentilla anserina* and four subgenomes of *F. ananassa* were used for calculating the non-synonymous (Ka) and synonymous (Ks) values. The BLASTP was to identify syntenic gene pairs of *P. anserina* and subgenomes separately. The Ka and Ks value of each syntenic gene pair with the syntenic file generated by MCScanX [105]. The Ka/Ks values were plotted by the R package.

### Phasing the subgenomes of ‘Benihoppe’ assembly and inference the phylogeny tree between *Fragaria* genomes

We used SubPhaser [60] (with default parameters) to phase the subgenomes of ‘Benihoppe’ assembly. To detect the sequences alignment between subgenomes and released *Fragaria* genomes, DNA and protein sequences including diploid strawberries (*F. vesca*, *F. nipponica*, *F. iinumae*, *F. nubicola*, *F. viridis*, *F*. *nilgerrensis*, *F. pentaphylla*, *F. daltoniana*) and *Potentilla micrantha* (outgroup) were downloaded from the GDR database (https://www.rosaceae.org/species/fragaria/all). Orthofinder (v.2.3.1) [106] was used to identify single-copy genes with the parameters ‘-M msa -S diamond’. The single-copy gene sequences were aligned with MAFFT (v7.487) and trimmed with trimAL (v1.2) [107]. Then maximum likelihood (ML) phylogenetic tree was inferred using IQ-TREE (v1.6.12) [108] with 1000 bootstraps. For the coalescence-based method, the gene trees were input into ASTRAL (v5.7.8) to infer the tree based on coalescence. Whole genome alignments were conducted using nucmer program in MUMmer 4 [109]. We generated synteny and structural rearrangements between two haplotype genomes using the pipeline for genome difference visualization ([110] https://github.com/schneebergerlab/plotsr).

### Gene expression analysis during strawberry fruit ripening

The three stages (green stage, intermediate red stage, and full red stage) of cultivar strawberry ‘Benihoppe’ fruit was used for gene expression analysis [46]. The published datasets (accession number GSE113084) were downloaded. Raw RNA-seq reads were trimmed by quality using Trimmomatic (v.0.39) [111]. HISAT2 (v.2.2.1) [112] was used to mapping the reads. FeatureCounts (v.2.0.1) [113] was used to calculated raw counts.. We used JCVI pipeline (https://github.com/tanghaibao/jcvi) to detect the homeologs gene pairs between A and the other three subgenomes, i.e., B, C, D, respectively. The first method is to define the differentially expressed homeologs gene pairs (DEGs) by directly comparing the transcript numbers between each homeologs gene pairs. The second method is to define DEGs using edgeR [69]. We finally characterized the biological function of hemizygous genes. The function of protein sequences was annotated using eggnog-mapper (http://eggnog-mapper.embl.de/) [114]. GO analysis was performed using the ClusterProfiler package [115] in R 4.1.0. *P* value <0.05 represented significantly enriched terms.

### Methylation analysis

Whole-genome bisulfite sequencing data (SRR6995966) of ‘Hongjia’ (also known as ‘Benihoppe’) leaves was downloaded from NCBI SRA database. The methylation data was analysed according to the method [46]. The cleaned reads were mapped to each subgenome of ‘Benihoppe’ assembly with bismark [116]. A minimum coverage of two was required at each cytosine to determine methylation status. The deepTools [117] was used to perform DNA methylation distribution plots. All methylation contexts were located within 1 kb upstream of the transcription start site (TSS) and 1 kb downstream of the transcription end site (TES).

### Inferences of gene body methylation

For each gene, the methylation state was inferred using a similar strategy [70, 118] Briefly, we used a binomial test to assess whether coding regions had a significantly higher proportion of methylated cytosines than the genome-wide background level of coding regions methylation [119]. This was performed for each cytosine context (CG, CHG, and CHH) separately. *P*-values were corrected for multiple tests using the Benjamini and Hochberg correction for each accession separately.

## Acknowledgements

This study was supported by grants from the National Key Research and Development Program (2022YFD1600700, 2019YFD1000203), the Major Science and Technology Projects of Henan Province (221100110400), the Special Fund for Henan Agriculture Research System (HARS-22-09-G2), and the Agricultural Science and Technology Innovation Program (CAAS-ASTIP-2021-ZFRI).

## Author contributions

H.Z and Y.Z. supervised and managed the project. Y.S. performed the bioinformatic analysis and organized and wrote the manuscript. Y.P. performed the methylation analysis and advised on data interpretation. L.L., G.L., and X.Z. performed tissue culture of strawberry in the germplasm nursery. X.W., S.C., and A.M. participated in the data analysis. All authors approved the submitted version.

## Data availability

All PacBio sequence data and genome assembly have been deposited to the NCBI short reads achieved under the project number: PRJNA966915 and PRJNA970713.

## Conflict of interest statement

The authors declare no conflicts of interest.

## Supplementary data

Supplementary data is available at *Horticulture Research* online.

## Supplementary Material

Web_Material_uhad252
