# Supplementary material for: Phased gap-free genome assembly of octoploid cultivated strawberry illustrates the genetic and epigenetic divergence among subgenomes
Source: Hortic Res. 2023 Nov 27;11(1):uhad252. doi: 10.1093/hr/uhad252 (PMC10807706; doi:10.1093/hr/uhad252)
Supplement: Web_Material_uhad252 [file web_material_uhad252.zip › Revised_Benihoppe supplement figures.docx]

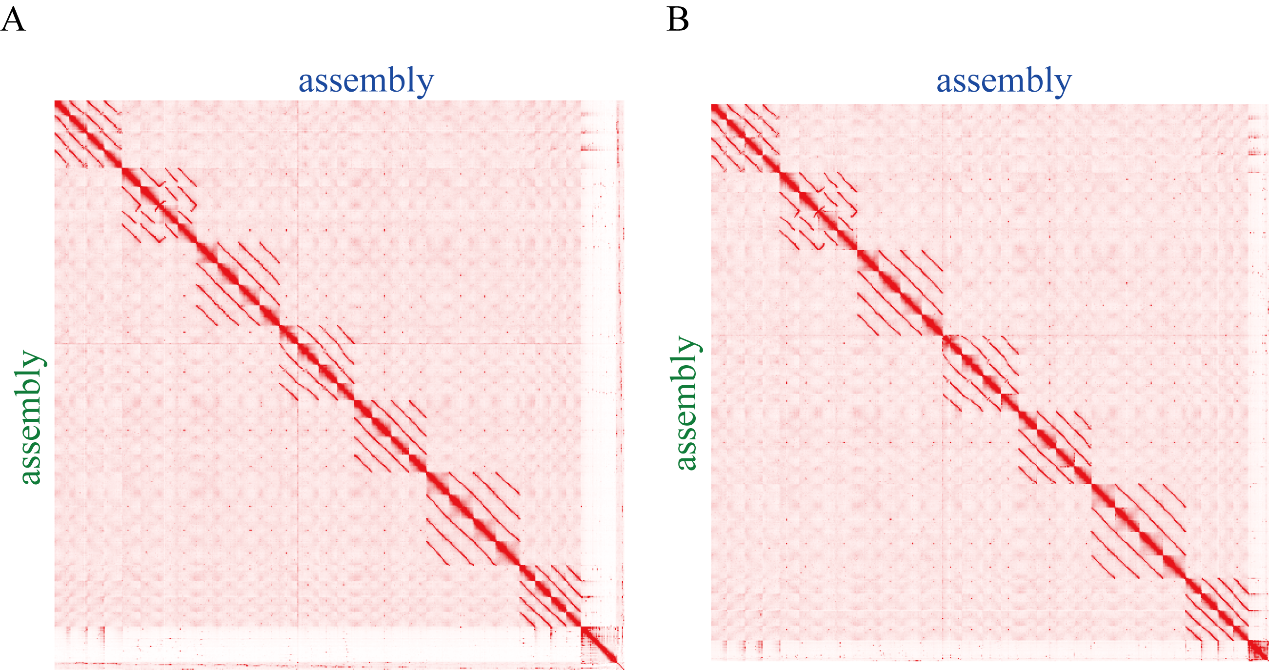


Figure S1 Genome-wide high-throughput chromosome conformation capture (Hi-C) interaction heatmap for the 28 linkage groups in haplotype 1 (A) and haplotype 2 (B) of ‘Benihoppe’ genome.


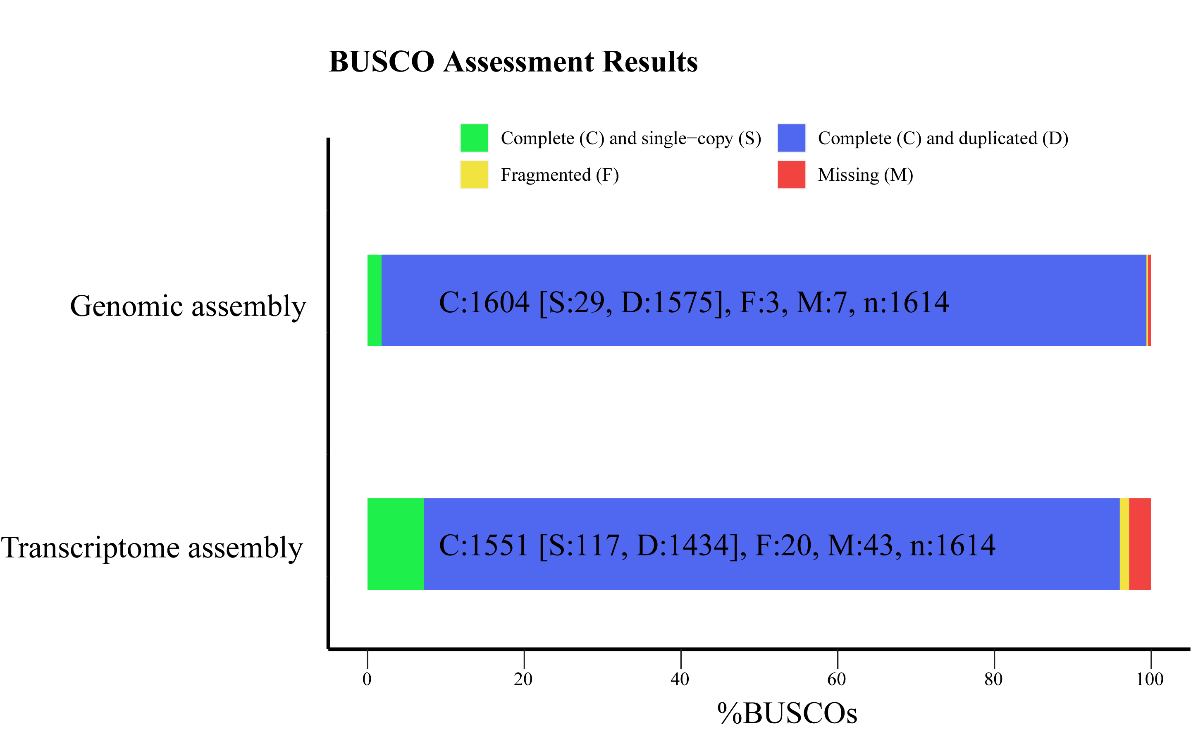


Figure S2 Benchmarking Universal Single-Copy Orthologs (BUSCO) analysis of genome assembly and annotation completeness of ‘Benihoppe’.


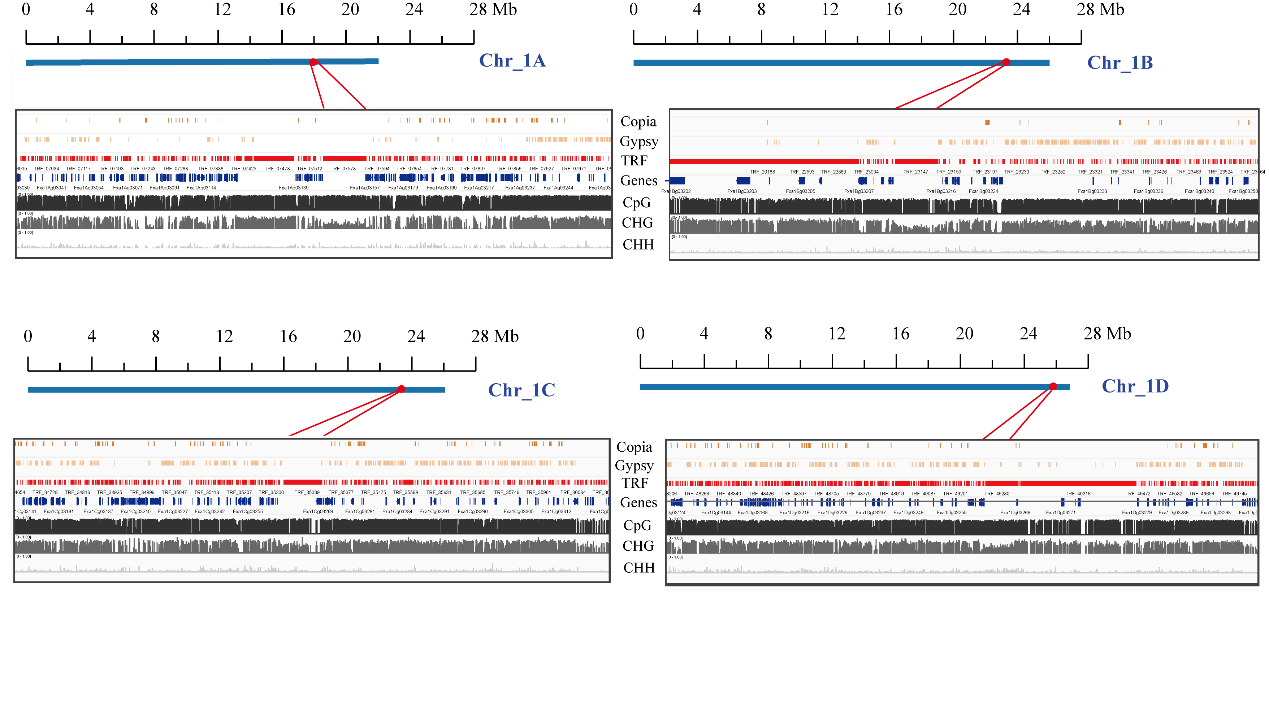


Figure S3 Distribution of TEs (Copia and Gypsy), genes, and DNA methylation in the CG, CHG, and CHH contexts around centromeric regions of FaChr1. Tandem Repeats Finder (TRF) output is shown in red.


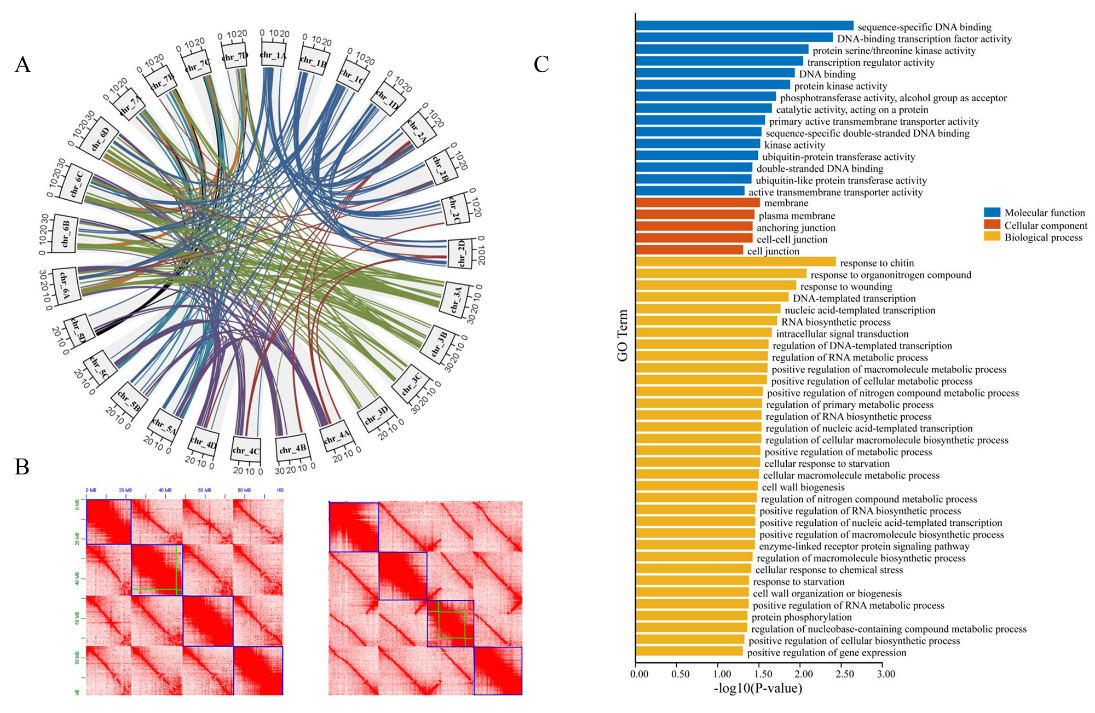


Figure S4 The chromosome structure for ‘Benihoppe’ genome. (A) Sequence alignments of all chromosomes of haplotype1. Different colors show some sequence rearrangements on the whole genome. (B) Hi-C heat map analysis showing chromosome structure variation on chr_1A, 1B, 1C, and 1D (left) and chr_2A, 2B, 2C, and 2D (right). Red blocks represent inversion and translocation. (C) Gene ontology (GO)-term enrichment of the duplicated genes in chromosomes 3 and 6.


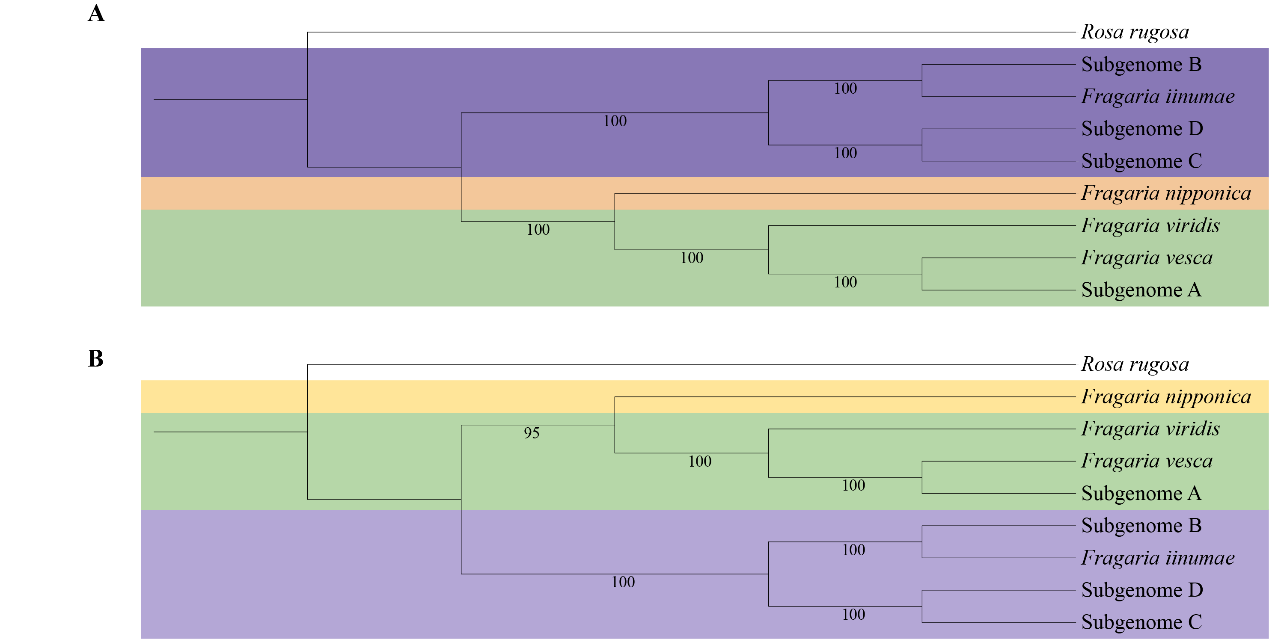


Figure S5 The maximum likelihood (ML) phylogenetic tree among the ‘Benihoppe’ subgenomes and four diploid genomes. (A)The ML tree was inferred by IQ-TREE with 1000 bootstraps. (B) The phylogenetic tree based on coalescence gene tree using ASTRAL.


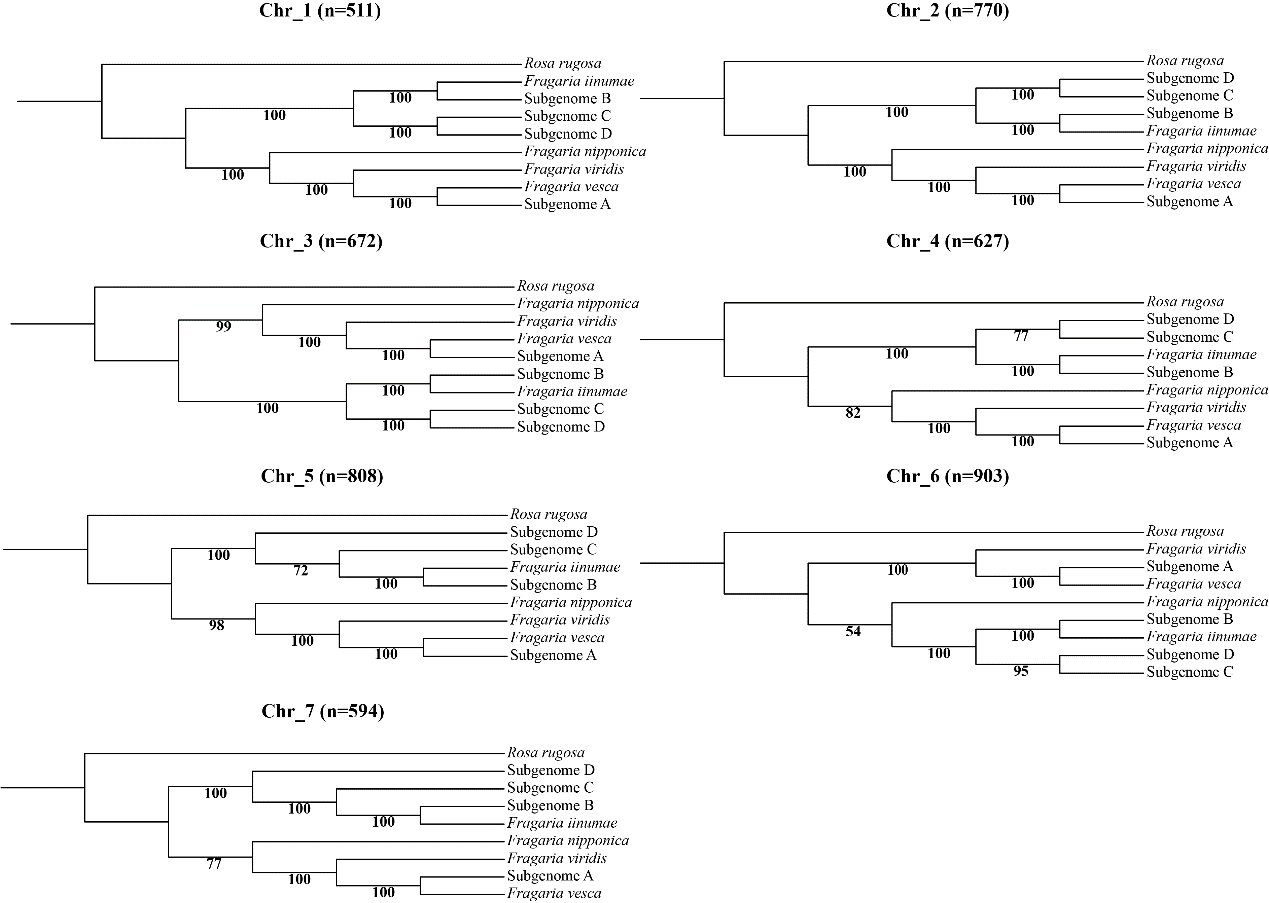


Figure S6 The phylogenetic tree inferred from chromosome 1 to chromosome 7 on single-copy genes. The numbers of single-copy genes are shown in each chromosome. The maximum-likelihood tree was reconstructed using IQ-TREE with 1000 bootstraps.


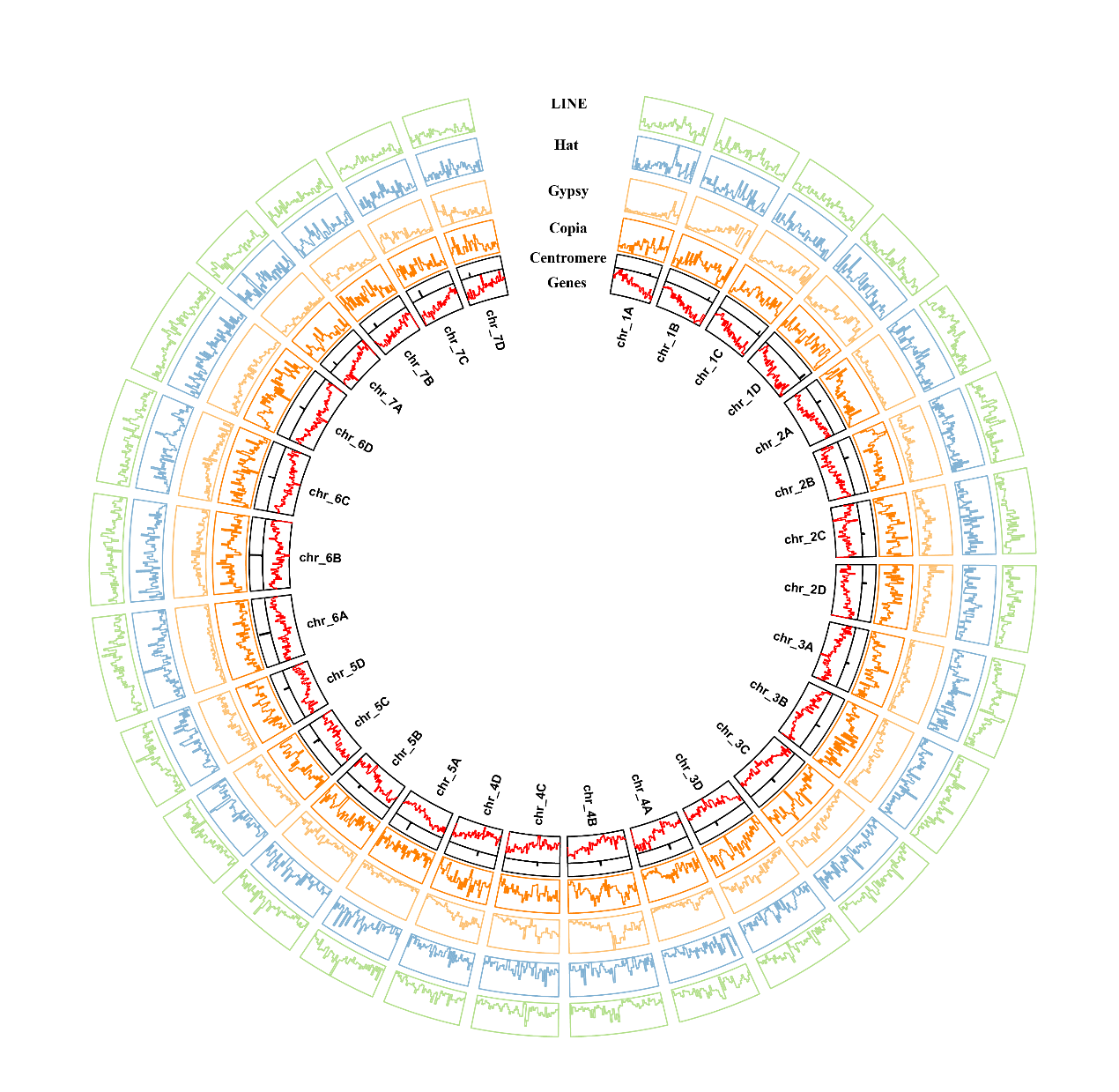


Figure S7 Repetitive sequences identified using RepeatMasker in each subgenome. Genomic feature distribution in the octoploid strawberry genome. The tracks from the inside out represent gene density per megabase, centromere location, Copia, Gypsy, Hat, and LINE density per megabase across each chromosome.


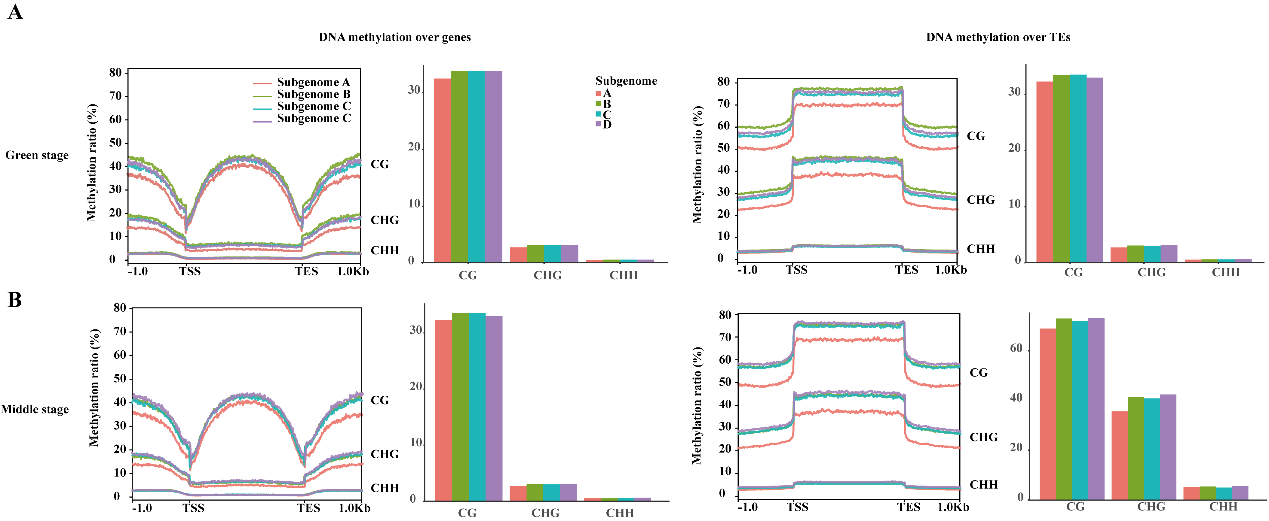


Figure S8 Genome-wide DNA methylation patterns over genes and TEs at the green and middle stages.


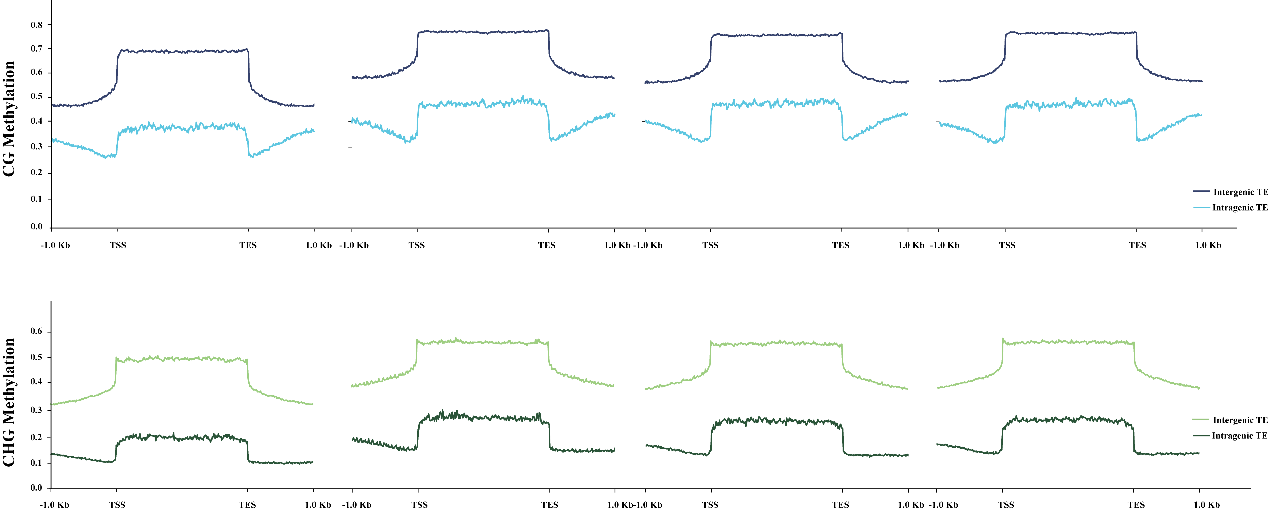


Figure S9 DNA methylation levels on CG and CHG contexts over intragenic TE and intergenic TE.


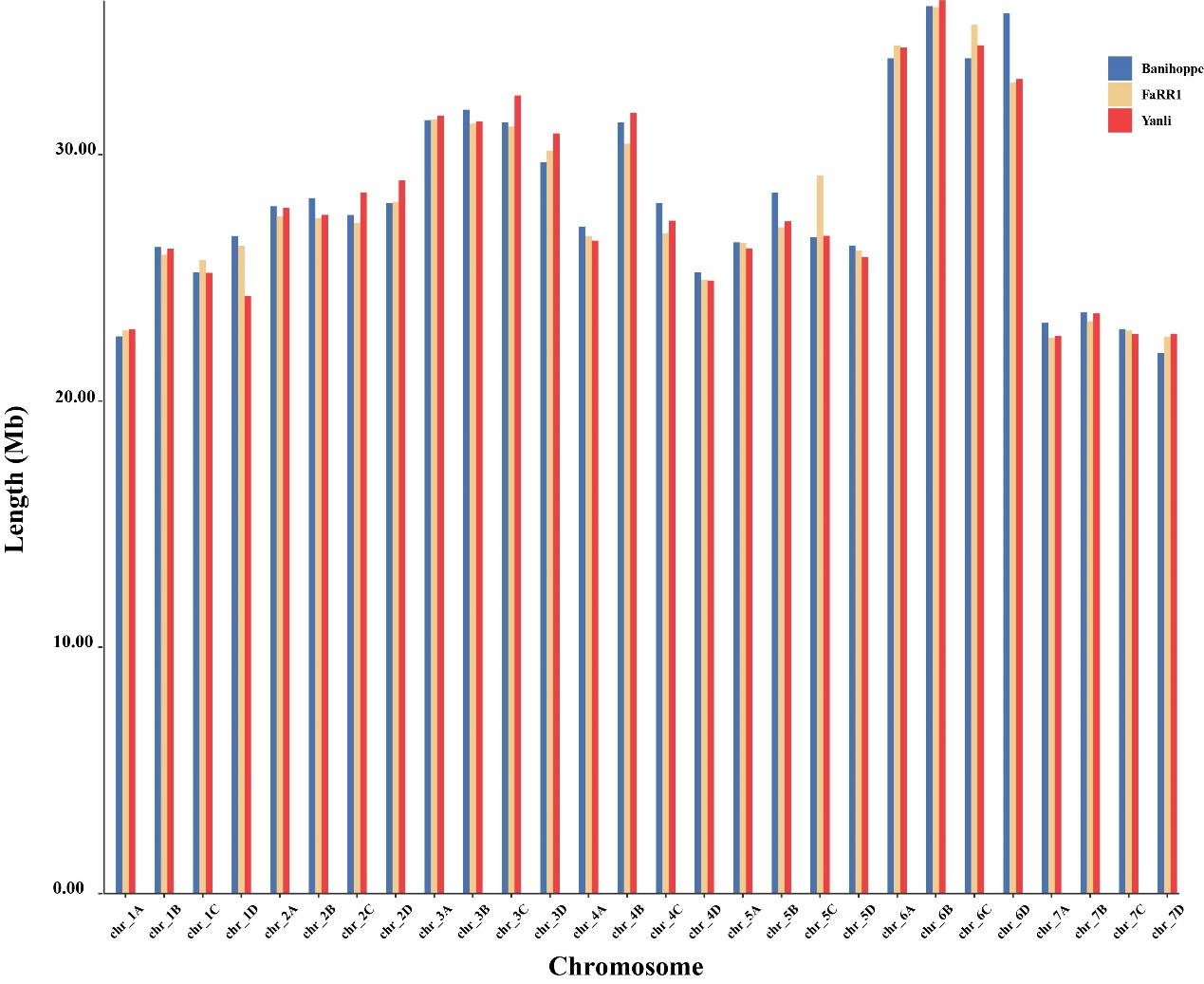


Figure S10 The lengths of chromosomes of ‘Benihoppe’, ‘FaRR1’, and ‘Yanli’.


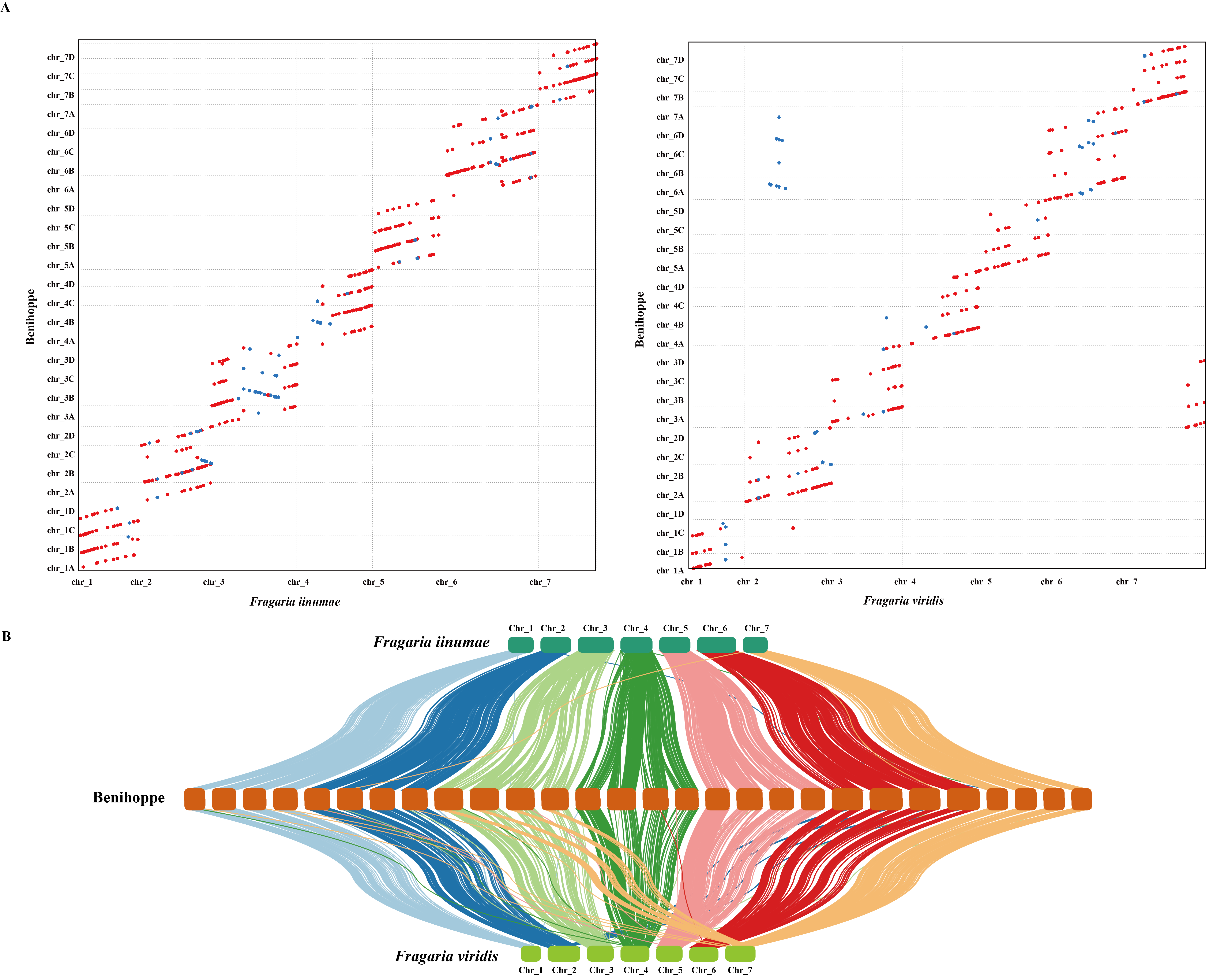


Figure S11 Whole genome alignments between ‘Benihoppe’ assembly and diploid genomes. (A) Dotplot of ‘Benihoppe’ assembly and *Fragaria iinumae* (left)/ *Fragaria* *viridis* (right); (B) Genome syntenic relationships across multiple genomes. The orange boxes indicate the 28 chromosomes of ‘Benihoppe’ assembly.
